# Silencing of PSMC2 inhibits development and metastasis of prostate cancer through regulating proliferation, apoptosis and migration

**DOI:** 10.1186/s12935-021-01934-8

**Published:** 2021-04-26

**Authors:** Qingke Chen, Lingmin Fu, Jieping Hu, Guanghua Guo, An Xie

**Affiliations:** 1 Department of Urology, First Affiliated Hospital of Nanchang University, Nanchang, China; 2 Institute of Urology, First Affiliated Hospital of Nanchang University, 17 Yong Wai Zheng Street, Nanchang, Jiangxi China; 3 Department of Burns, First Affiliated Hospital of Nanchang University, Nanchang, China; 4 Jiangxi Health Vocational College, Nanchang, China

**Keywords:** Prostate cancer, PSMC2, Cell proliferation, Cell apoptosis, Akt pathway

## Abstract

**Background:**

Prostate cancer is the most common malignant tumor of male genitourinary system, molecular mechanism of which is still not clear. PSMC2 (proteasome 26S subunit ATPase 2) is a key member of the 19S regulatory subunit of 26S proteasome, whose relationship with prostate cancer is rarely studied.

**Methods:**

Here, expression of PSMC2 in tumor tissues or cells of prostate cancer was detected by qPCR, western blotting and immunohistochemical analysis. The effects of PSMC2 knockdown on cell proliferation, colony formation, cell migration, cell cycle and apoptosis were assessed by Celigo cell counting assay, colony formation assay, wound-healing assay, Transwell assay and flow cytometry, respectively. The influence of PSMC2 knockdown on tumor growth in vivo was evaluated by mice xenograft models.

**Results:**

The results demonstrated that PSMC2 was upregulated in tumor tissues of prostate cancer and its high expression was significantly associated with advanced Gleason grade and higher Gleason score. Knockdown of PSMC2 could inhibited cell proliferation, colony formation and cell migration of prostate cancer cells, while promoting cell apoptosis and cell cycle arrest. The suppression of tumor growth in vivo by PSMC2 knockdown was also showed by using mice xenograft models. Moreover, the regulation of prostate cancer by PSMC2 may be mediated by Akt/Cyclin D1/CDK6 signaling pathway.

**Conclusions:**

Therefore, our studies suggested that PSMC2 may act as a tumor promotor in the development and progression of prostate cancer, and could be considered as a novel therapeutic target for prostate cancer treatment.

## Background

Prostate cancer is the most common malignant tumor of male genitourinary system [1]. In United States, prostate cancer has been identified as the most common malignant tumor in men, accounting for 19% of morbidity of male cancer, which is also the second most fatal cancer in men, accounting for about 9% of all cancer-related deaths [2]. The statistical data showed that, in 2018, there were 164,690 new cases of prostate cancer and 29,430 deaths from prostate cancer in United States [2–4]. Although the progression of prostate cancer is relatively slow and radical treatment has exhibited excellent therapeutic effects on early and localized prostate cancer, the tumors of a large number of prostate cancer patients have progressed to advanced stage when diagnosed who can only be treated by endocrine therapy [5–8]. However, after endocrine therapy, most prostate cancer patients will progress to castration-resistant prostate cancer, which is insensitive to chemotherapy and radiotherapy, thus suffering poor prognosis due to the lacking of effective therapy and the tendency of metastasis [9–11]. Therefore, as a prerequisite for the development of novel treatment strategies for prostate cancer, it is of great urgency to deepen the understanding of the molecular mechanism of the development and metastasis of prostate cancer [12–14].

Previous studies have showed that 26S proteasome participated in the regulation of a variety of biological processes such as cell cycle progression [15], apoptosis [16], metabolic regulation [17], and signal transduction [18]. With the continuous exploration of 26S proteasome, its substrates have attracted considerable attention. PSMC2 (proteasome 26S subunit ATPase 2), located in 7q22.1-q22.3 of the genome, is a key member of the 19S regulatory subunit of 26S proteasome, responsible for catalyzing the unfolding and translocation of substrates into the 20S proteasome [19]. In addition, because of the intracellular existence of free 20S granules, but not 19S granules, the 26S proteasome assembly is limited by the level of 19S regulatory subunit [20]. Therefore, the expression of PSMC2 may be necessary for the assembly of 19S and 26S proteasomes. Nijhawan et al*.* listed PSMC2 as the highest-ranked gene of CYCLOPS (Copy-number alterations Yielding Cancer Liabilities Owing to Partial losS), which represents a special subset of essential genes related to the activity of cancer cells. It was demonstrated that some genomic deletions of PSMC2 were observed in more than 3000 tumors, which made the cancer cells highly dependent on the remaining PSMC2, and further indicated that PSMC2 could be used as a potential target for cancer treatment [20]. Previous studies have also confirmed the important role of PSMC2 in some human cancers [21]. For example, the expression of PSMC2 increased in tumors of p21-HBx transgenic mice and downregulation of PSMC2 inhibited the proliferation of ovarian cancer cells [22]. Moreover, the role of PSMC2 in the development and progression of osteosarcoma has also been revealed as a tumor promotor [23, 24]. Despite that PSMC2 is considered to be a newly discovered gene closely related to human cancer, its relationship with prostate cancer is still unclear.

The emphasis of this study lied in a potential promotor acted by PSMC2 in the development and metastasis of prostate cancer. PSMC2 expression significantly increased in prostate cancer tissues and cell lines. The behavior of the correlation coefficient made us conclude that a positive relationship between PSMC2 overexpression with advanced Gleason grade and higher Gleason score. Furthermore, the inhibition of PSMC2 led to the decrease in prostate cancer cell proliferation, colony formation and cell migration, while facilitated cell apoptosis of prostate cancer cells. Also, PSMC2 knockdown exerted its tumor suppressive function in vivo. Moreover, the involvement of PSMC2 in prostate cancer was mediated by the activity of Akt/Cyclin D1/CDK6 signaling pathway. As such, this study provided novel insights into PSMC2 as a potential therapeutic target for the treatment of prostate cancer.

## Methods

### Cells and antibodies

Human prostate cancer cells PC-3 and DU 145 were purchased from the Cell Bank of Typical Culture Preservation Committee of Chinese Academy of Sciences (Shanghai, China) and C4-2 were obtained from ATCC (Manassas, VA, USA). PC-3 cells were cultured in F-12 medium (Gibco, Rockville, IN, USA), DU 145 were kept in MEM medium (Gibco, Rockville, IN, USA) and C4-2 were maintained in 400 mL MEM (Lonza, Basel, Switzerland) plus 100 mL F12 Medium. All the medium was supplemented with 10% fetal bovine serum (FBS) and all cultured medium changed every 3 days. All cells were maintained at 37˚C with 5% CO_2_ and 95% humidity.

Antibodies used in our study were PSMC2 (Cat # SC-166972, Santa Cruz, CA, USA), GAPDH Rabbit (Cat #AP0063, Bioworld, St. Louis, MN, USA), HRP goat anti-rabbit/mouse IgG (Cat # A0208/ A0216, Beyotime, Beijing, China), Akt and Cyclin D1 (Cat #4685/2978, CST, Danvers, MA, USA), p-Akt (Cat #bs-5193r, Bioss, Beijing, China), CDK6 (Cat #ab151247, Abcam, Cambridge, MA, USA), P21 (Cat #BM3990, Boster, Wuhan, China), Ki67 (Cat #ab16667, Abcam, Cambridge, MA, USA) and HRP goat anti-rabbit IgG (Cat #ab151247, ab40776, ab76125, ab16667 and ab6721, Abcam, Cambridge, MA, USA).

Akt inhibitor MK-2206 was purchased from MedChemExpress (Cat #HY-10358).

### Immunohistochemistry

Prostate cancer tissue microarray chip was obtained including 152 cases of prostate cancer tissues and 80 cases of normal prostatic tissues. Patients’ information and related data were collected as well. Written informed consent was provided by each patient before the operation. Our study protocol was approved by Ethics Committee of First Affiliated Hospital of Nanchang University. For immunohistochemistry, tissue slides were bake at 65 °C for 30 min in oven. After dehydrated in xylene and rehydrated in 100% and 75% alcohol, EDTA was added for antigen retrieval in 100 °C boiling water for 30 min. After washing with 1 × PBS + 0.1%Tween20, slides were blocked with 3% H_2_O_2_ for 5 min. Primary antibody specific to PSMC2 were added for incubating at 4 °C overnight. Then the second antibody HRP goat anti-rabbit IgG was added and incubated for 1 h at 37 °C. All slides were wash and DAB color with DAB dye solution for 5 min without light, then all slices were counterstained with hematoxylin. Slides were pictured with microscopic 200 × and 400 × Objectives and viewed by ImageScope and CaseViewer. All slides were examined by two independent pathologists. Staining percentage scores were classified as: 1 (1–24%), 2 (25–49%), 3 (50–74%) and 4 (75–100%). Staining intensity were scored as 0 (Signalless color), 1 (brown), 2 (light yellow), 3 (dark brown).

### Plasmid construction and stable transfection

For overexpression, the PSMC2 construct was produced by subcloning human PSMC2 cDNA into vector BR-V-208 (Shanghai Biosciences, Shanghai, China). For knockdown, three small hairpin RNAs (shRNA) of PSMC2 was synthesized (5′-GCCAGGGAGATTGGATAGAAA-3′, 5′-CAACGTAAAGCAGTTTGCCAA-3′, 5′-AAGCAAGTTGAAGATGACATT-3′) and cloned into BR-V108 vector. Plasmids were collected and purified using EndoFree Maxi Plasmid Kit and transfected into 293T cells to package lentivirus.

DU 145 and PC-3 cells were transfected with the packaged lentivirus along with ENI.S and Polybrene additives. After 72 h culturing, fluorescence and cell infection efficiency was observed and valued by microscopic.

### RNA isolation and RT-PCR analysis

Transfected human prostate cancer cells were fully lysed with Trizol (Sigma, St Louis, MO, USA). The concentration and quality of extracted RNA was determined by Nanodrop 100 Spectrophotometer (Thermo Fisher Scientific, Waltham, MA, USA). Total RNA was converted to cDNA with ReverAid First Strand cDNA kit (Thermo Fisher Scientific, Waltham, MA, USA) according to the manufacturer's recommendation. RT-PCR was performed with the Qiagen One-step RT-PCR kit (Vazyme, Nangjing, Jiangsu, China). PCR procedures are as follows: 95 °C for 70 s, 60 °C for 30 s, 95 °C for 30 s, 95 °C for 15 s, 55 °C for 60 s and 95 for 15 s, 45 cycles. Primers to anneal to PSMC2 were 5′-CAGCACTCTGGGATTTGGCT-3′ and 5′-TTTCTATCCACGCCCACTCTC-3′ and primers to anneal to inner control GAPDH were 5′-TGACTTCAACAGCGACACCCA-3′ and 5′-CACCCTGTTGCTGTAGCCAAA-3′.

### Western blot and human apoptosis antibody array-membrane

Cells were washed with cold PBS and lysed in ice-cold lysis buffers containing Western, IP lysate and PMSF. Total protein concentrations were determined using the BCA method with BCA Protein Assay Kit (HyClone-Pierce, Logan, UT, USA). Proteins (20 μg per lane) were separated by electrophoresis on a 10% SDS-PAGE gel, and transferred to nitrocellulose membranes. The membranes were blocked with TBST solution containing 5% skimmed milk for 1 h. Primary antibodies specific to Akt (1:1000), p-Akt (1:1000), Cyclin D1 (1:2000), CDK6 (1:1000), p21 (1:500) were added and incubated at 4 °C overnight. Then the membranes were incubated with horseradish peroxidase-conjugated goat anti-rabbit secondary antibodies (1:3000) for 1 h at room temperature. Western Chemiluminescent HRP Substrote kit was used for coloring (Millipore, Schwalbach, Germany). GAPDH served as the internal standard and the blot bands were visualized with enhanced chemiluminescence (ECL) (Amersham, Chicago, IL, USA) system.

For human apoptosis antibody array, total protein from was PC-3 in shCtrl and shPSMC2 groups were collected after fully lysed by lysis buffer. Protein samples were added for incubating with blocked array antibody membrane overnight at 4 °C. After washing, 1:100 Detection Antibody Cocktail was added incubating for 1 h, followed by incubated with HRP linked streptavidin conjugate for 1 h. All spots were visualized by enhanced chemiluminescence and the signal densities were analyzed with ImageJ software (National Institute of Health, Bethesda, MD, USA).

### Celigo cell counting assay

Lentivirus-infected (shCtrl, shPSMC2) DU 145 and PC-3 cells were seeded at a 96-well plate with 2000 cells per well for culturing. The plate was continuously detected by Celigo (Nexcelom, Lawrence, MA, USA) for 5 days at the same time. Cell proliferation rate was analyzed.

### MTT assay

Cells were seeded on 96-well plates. After trypsinization, culture medium was resuspended into cell suspension. Cell density was adjusted to 2000 cells/well and inoculated to 96-well plates (100 µL/well) (Corning, Corning, NT, USA, #3599). MTT (3-(4, 5-dimethylthiazol-2-yl)-2, 5-diphenyl tetrazolium bromide) (Genview, Beijing, China; # JT343) solution was added per well for 4 h. After removing the culture medium containing MTT and adding dimethyl sulfoxide (DMSO), the cells were shaken for 10 min at room temperature. The absorbance was measured at 490 nm with Microplate Reader (Tecan) and the cell viability was calculated.

### Cell apoptosis assay

Lentivirus-infected (shCtrl, shPSMC2) DU 145 and PC-3 cells (1 × 10^3^ cells/mL) were inoculated in a 6-well plate with 2 mL per well and further cultured for 5 days. Cells were collected with centrifugation at 1200×*g*, then cells were resuspended with binding buffer, then 10 μL Annexin V-APC (eBioscience, San Diego, CA, USA) was added for staining without light. Apoptosis analyses was measured using FACSCalibur (BD Biosciences, San Jose, CA, USA).

### Colony formation assay

Lentivirus-infected (shCtrl, shPSMC2) DU 145 and PC-3 cells in the logarithmic growth phase were collected, digested and resuspended. Cells were seeded in a 6-well plate with 1000 cells per well and cultured for 8 days, the culture medium was changed every 3 days. Colony photos were collected by fluorescence microscope (Olympus, Tokyo, Japan). After cells were fixed with 4% paraformaldehyde, Giemsa (Dingguo, Shanghai, China) was used for staining and the number of colonies (> 50 cells/colony) was counted.

### Wound healing assay

Lentivirus-infected (shCtrl, shPSMC2) DU 145 and PC-3 cells (5 × 10^4^ cells/well) were plated into a 96-well dish in triplicate for culturing. Scratches were made by a 96 wounding replicator (VP scientific, San Diego, CA, USA) across the cell layer with 90% confluence. Photographs were taken by a fluorescence microscope at 0 h, 24 h and 48 h after scratching. Cell migration rates of each group were calculated.

### Transwell assay

Lentivirus-infected (shCtrl, shPSMC2) DU 145 and PC-3 cells were incubated in the upper chamber with 100 μL medium without FBS in a 24-well plate (5 × 10^4^ cells/well). 600 μL medium supplemented with 10% FBS was added into the lower chamber. Cells were incubated for 24 h at 37 °C with 5% CO_2_. Finally, lower chamber cells were fixed by 4% formaldehyde and stained by Giemsa and cells from five random fields were selected for observing and the migration ability of cells was analyzed. Experiment was repeated in three wells.

### Nude mice xenograft tumor studies and HE staining

4 week-BALB/c nude mice were purchased from Shanghai Slake Laboratory Animal Co., Ltd. (Shanghai, China). Mice were maintained in SPF conditions and the experiment were approved by Ethics Committee of First Affiliated Hospital of Nanchang University. 0.2 mL (4 × 10^7^ mL D-Hanks) stably transfected PC-3 cells were subcutaneous injected into the nude mice, there were 10 mice in each group (shCtrl and shPSMC2 group). Mice weight and tumor sizes using W and L (W means width at the widest point of tumor; L means perpendicular width) were recorded two times per week 20 days post injection. After intraperitoneal (I.P.) injection of D-luciferin (15 mg/mL) at a dose of 10 μL/g, the mice were of 0.7% sodium pentobarbital for in vivo bioluminescence applying Lumina LT Xenogen VivoVision IVIS 100 system (Perkin Elmer, Waltham, MA, USA). 48 days later, all mice were euthanized and the tumor tissues were surgically dissected.

The tumor tissues were embedded in paraffin for hematoxylin and eosin (HE) staining. Before HE staining, slides were blocked with PBS-H_2_O_2_ and were incubated with primary antibody Ki-67 (1:200) at 4 °C overnight. Then slides were incubated with goat anti-rabbit IgG HRP (1:400) second antibody. Hematoxylin and Eosin (Baso, Zhuhai, Guangdong, China) were used for staining.

### Statistical analyses

All experiments were performed in triplicate. Data were shown as mean ± SD. The significance of the differences between groups was determined using the two-tailed Student’s t test or one-way ANOVA. Statistical significance was calculated by SPSS 22.0 (IBM, SPSS, Chicago, IL, USA), and *P* < 0.05 was considered statistically significant. Graphs were made using GraphPad Prism 6.01 (Graphpad Software, La Jolla, CA, USA).

## Results

### PSMC2 is upregulated in cancer of the prostate

**Fig. 1:**
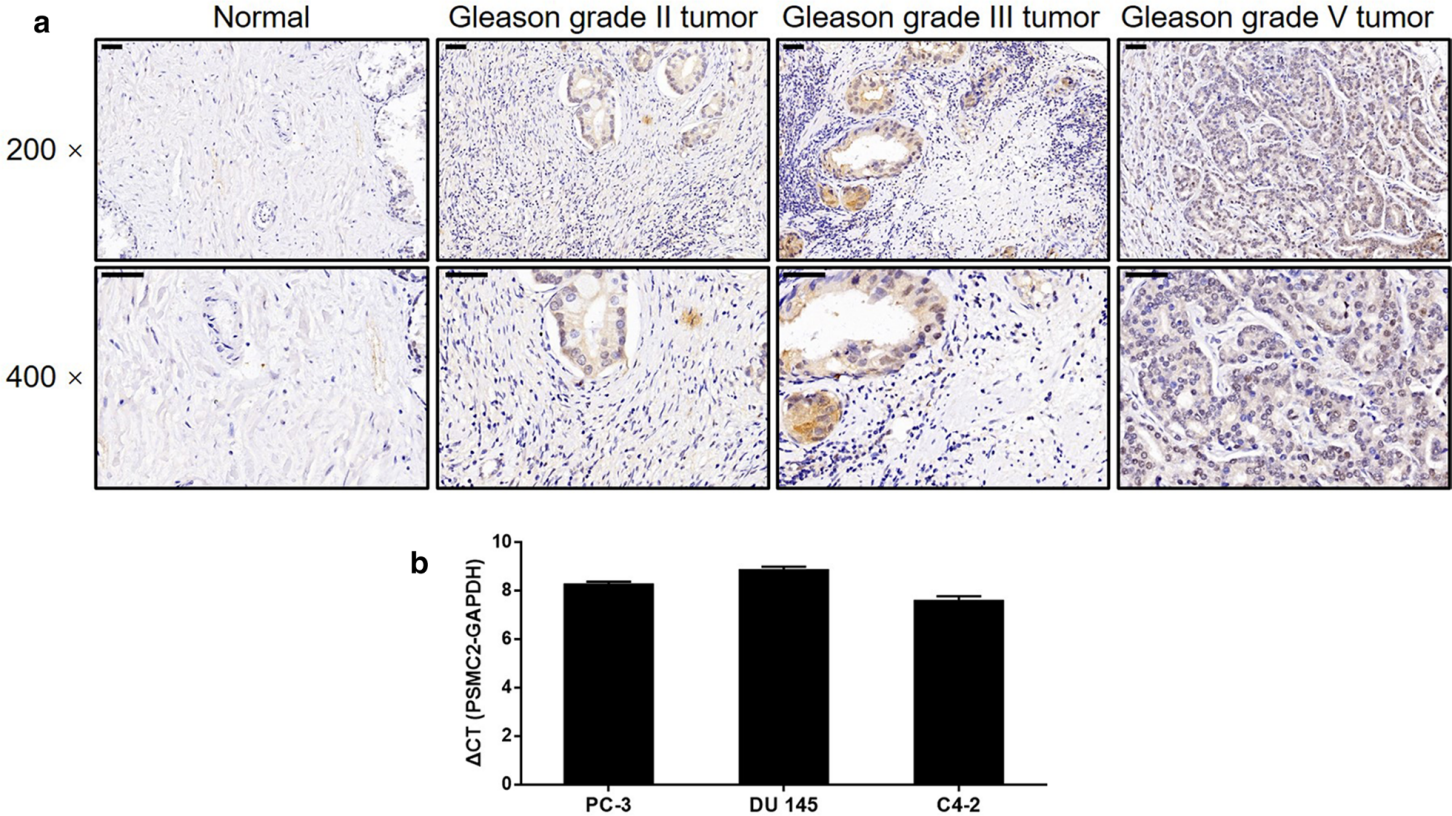
PSMC2 was upregulated in prostate cancer. **a** The expression of PSMC2 in prostate cancer tumor tissues with different tumor stages and normal tissues was detected by IHC (magnification ×200/×400). **b** The background expression of PSMC2 in prostate cancer cell lines was detected by qPCR. Results were presented as mean ± SD

Immunohistochemical (IHC) analysis was used to investigate the expression levels of PSMC2 in specimens of tumor acquired from 145 patients with prostate cancer; these data were then compared with the expression levels of PSMC2 in normal control tissues. It was clear from the resulting data that the expression of PSMC2 was clearly upregulated in tumors (Table 1, Fig. 1a). It was also evident that tumors with a higher Gleason grade expressed higher levels of PSMC2, thus indicating a positive relationship between PSMC2 levels and Gleason grade in cases of prostate cancer (Fig. 1a). There was a significant correlation between the expression of PSMC2 and the Gleason score and grade (Table 2), as confirmed by Spearman’s rank correlation analysis (Table 3). Collectively, these results clarified the potential role of PSMC2 as a tumor promoter in the development and progression of prostate cancer. qPCR further detected high levels of PSMC2 expression in a range of prostate cancer cell lines (DU 145, PC-3, and C4-2) thus demonstrating the strong association between PSMC2 expression and prostate cancer (Fig. 1b).

**Table 1:** Expression patterns of PSMC2 in prostate tissues and normal tissues revealed in immunohistochemistry analysis

PSMC2 expression	Tumor tissue		Normal tissue	
	Cases	Percentage	Cases	Percentage
Low	82	53.9%	77	96.3%
High	70	46.1%	3	3.7%

*P* < 0.001

**Table 2:** Relationship between PSMC2 expression and tumor characteristics in patients with prostate cancer

Features	No. of patients	PSMC2 expression		*P* value
		Low	High	
All cases	152	82	70	
Age (years)				0.272
≤ 69	79	46	33	
> 69	73	36	37	
Gleason score				0.031*
≤ 8	89	53	36	
> 8	56	23	33	
T infiltrate				0.420
T1	2	2	0	
T2	68	39	29	
T3	37	18	19	
T4	6	4	2	
Lymphatic metastasis (N)				0.735
N0	105	59	46	
N1	8	4	4	
Stage				0.077
I	14	13	1	
II	56	28	28	
III	31	16	15	
IV	12	6	6	
Gleason grade				< 0.001***
2	8	7	1	
3	38	26	12	
4	51	26	25	
5	47	17	30

**Table 3:** Relationship between PSMC2 expression and tumor characteristics in patients with prostate cancer analyzed by Spearman rank correlation analysis

Tumor characteristics	Index	
Gleason score	Pearson correlation	0.180
	Significance (two tailed)	0.030*
	n	145
Gleason grade	Pearson correlation	0.303
	Significance (two tailed)	< 0.001***
	n	145

### The silencing of PSMC2 led to the inhibition of proliferation and colony formation in prostate cancer cells

**Fig. 2:**
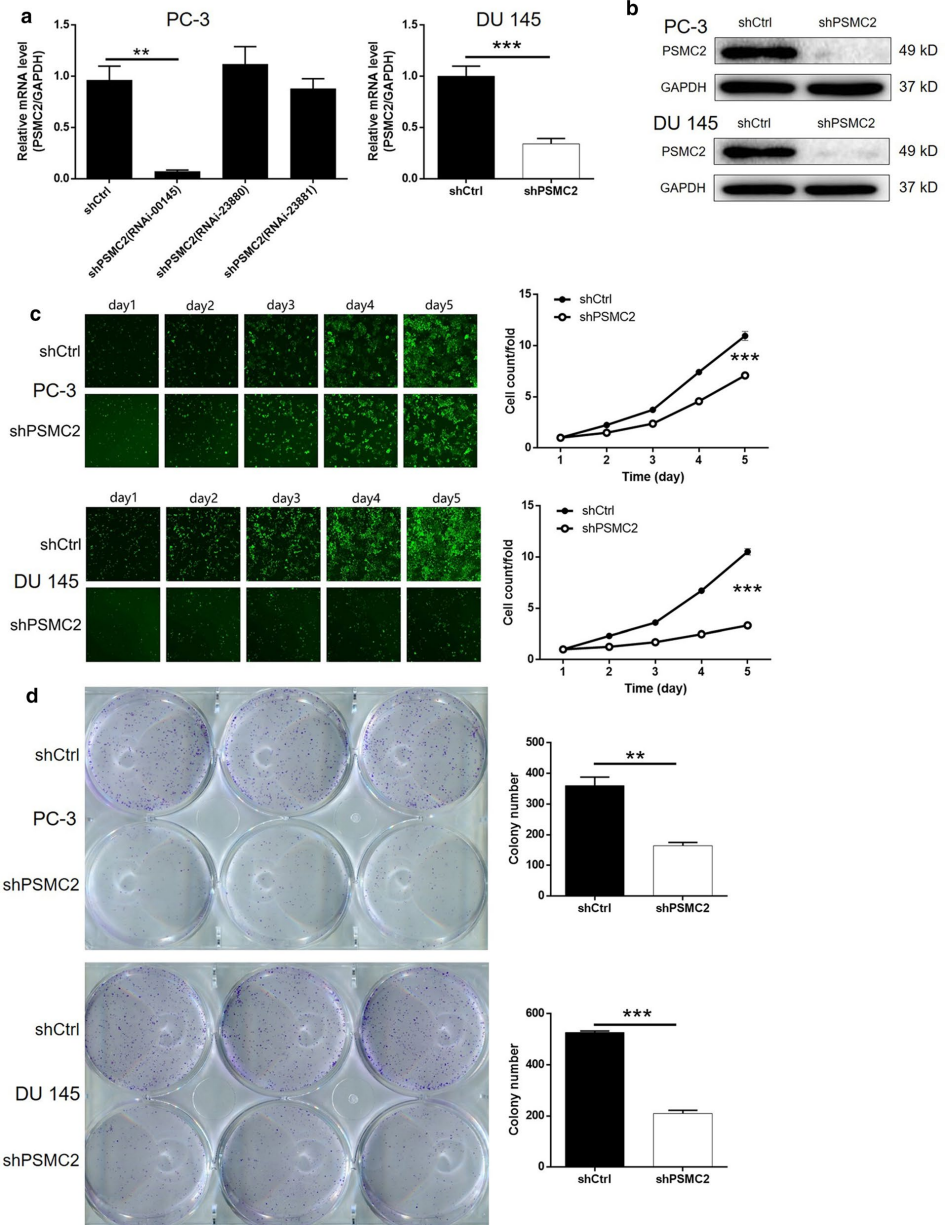
Knockdown of PSMC2 inhibited cell growth in vitro. PSMC2 knockdown cell models were constructed through the transfection of shCtrl or shPSMC2. **a** The knockdown efficiencies of PSMC2 in PC-3 and DU 145 cells were detected by qPCR. **b** The successful knockdown of PSMC2 in PC-3 and DU 145 cells were confirmed by western blotting. **c** The effects of PSMC2 knockdown on cell proliferation of PC-3 and DU 145 cells were evaluated by Celigo cell counting assay. **d** Colony formation assay was performed to detect the effects of PSMC2 on colony formation ability of PC-3 and DU 145 cells. Results were presented as mean ± SD. *P < 0.05, **P < 0.01, ***P < 0.001

Next, we aimed to investigate the specific effects of PSMC2 in cancer of the prostate. To facilitate this investigation, we designed three short-hairpin RNAs (shRNAs) that targeted PSMC2 (shPSMC2) and a negative control (shCtrl). These shRNAs were then packaged into a lentivirus vector alongside a green fluorescent protein (GFP) tag. These lentiviral vectors were subsequently transfected into two prostate cancer cell lines (PC-3 and DU 145) in order to construct a cell model for the knockdown of PSMC2. Observation of the signal arising from the GFP indicated that the efficiency of transfection in both cell types was > 80% (data not shown). Next, we used qPCR to determine the mRNA expression of PSMC2 in PC-3 cells in order to ascertain the efficiency of knockdown for the three shRNA constructs. It was evident that shPSMC2 (RNAi-00145) exhibited optimal effect (90% knockdown); this construct was therefore selected for use in subsequent experiments (Fig. 2a). We also verified the successful silencing of PSMC2 in PC-3 and DU 145 cells by both qPCR and western blotting (Fig. 2a and b). Celigo cell counting assays further showed that the silencing of PSMC2 led to a significant inhibition of cell proliferation in both PC-3 and DU 145 cells (*P* < 0.001, Fig. 2c). In addition, we also found that the knockdown of PSMC2 significantly weakened the abilities of PC-3 and DU 145 cells to form colonies; significantly fewer colonies were formed by cells in the shPSMC2 groups compared to the control groups over the same period of time (*P* < 0.01, Fig. 2d). Collectively, these results indicated that the PSMC2 knockdown inhibits the development and progression of prostate cancer by regulating mechanisms related to cell proliferation.

### Knockdown of PSMC2 promotes cell apoptosis and cycle arrest of prostate cancer cells

**Fig. 3:**
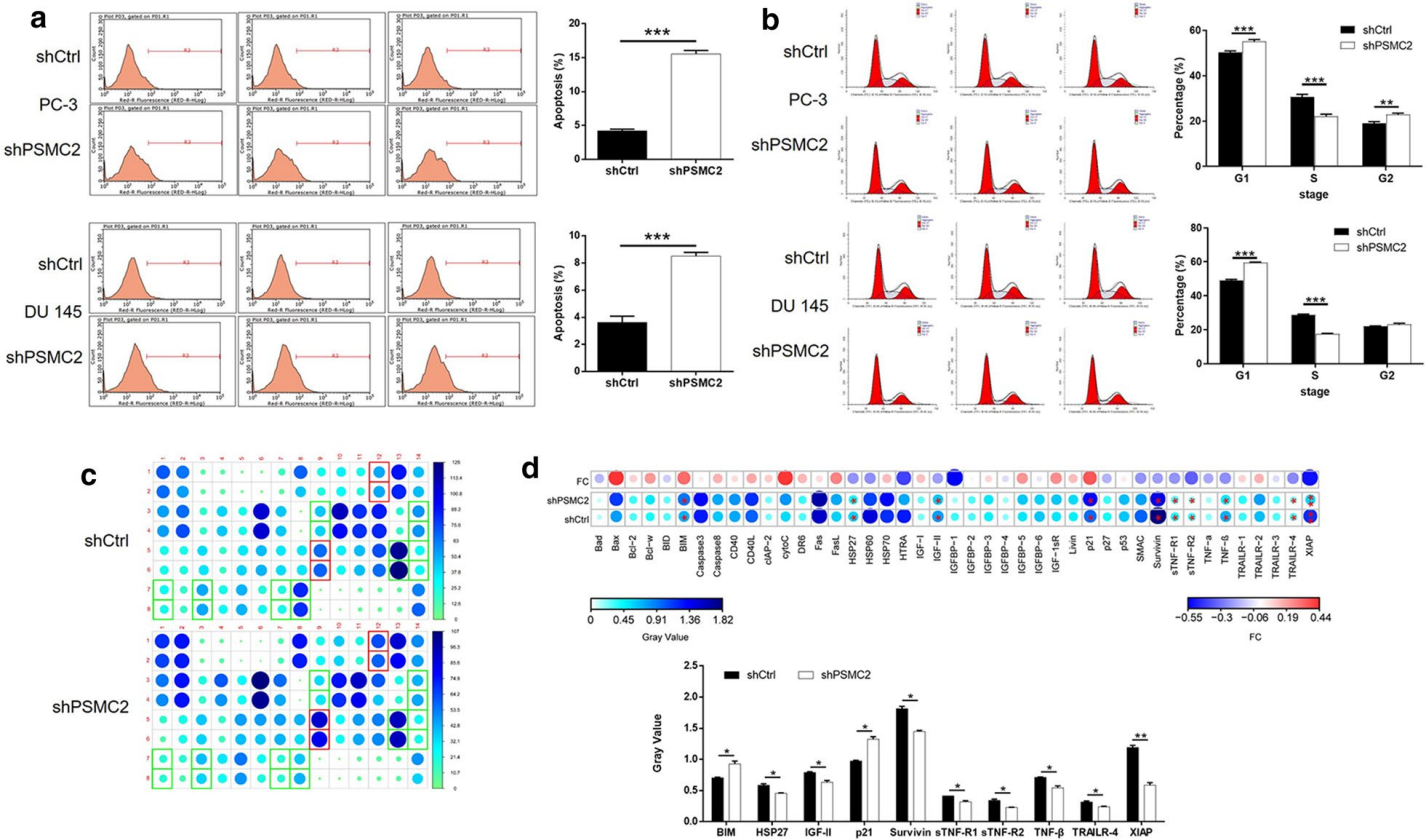
Knockdown of PSMC2 promoted cell apoptosis. **a**, **b** Flow cytometry was performed to examine the effects of PSMC2 knockdown on cell apoptosis (**a**) and cell cycle distribution (**b**) of PC-3 and DU 145 cells. **c**, **d** Human apoptosis antibody array was used to identify differentially expressed apoptosis-related proteins in PC-3 cells with or without PSMC2 knockdown. Results were presented as mean ± SD. *P < 0.05, **P < 0.01, ***P < 0.001

Next, we investigated the effects of PSMC2 knockdown on the proportion (%) of apoptosis in PC-3 and DU 145 cells by flow cytometry. Data indicated that the depletion of PSMC2 caused a significant increase in the proportion of cells showing evidence of apoptosis (*P* < 0.001, Fig. 3a). On the other hand, we also found that knockdown of PSMC2 induced the decrease of cell percentage in S phase and the increase of cell percentage in G1 phase, suggesting the arrest of cell cycle (Fig. 3b). In addition, the knockdown of PSMC2 also affected the expression of a range of proteins related to apoptosis, as determined by a Human Apoptosis Antibody Array (Fig. 3c). As shown in Fig. 3d, cells treated with shPSMC2 exhibited an upregulation of pro-apoptotic proteins (BIM and P21) and a downregulation of anti-apoptotic proteins (HSP27, IGF-II, Survivin, sTNF-R1, sTNF-R2, TNF-β, TRAILR-4, and XIAP) (*P* < 0.05). These results indicated that these factors are all involved in the PSMC2-induced regulation of cell apoptosis.

### The knockdown of PSMC2 inhibited the migration of prostate cancer cells

**Fig. 4:**
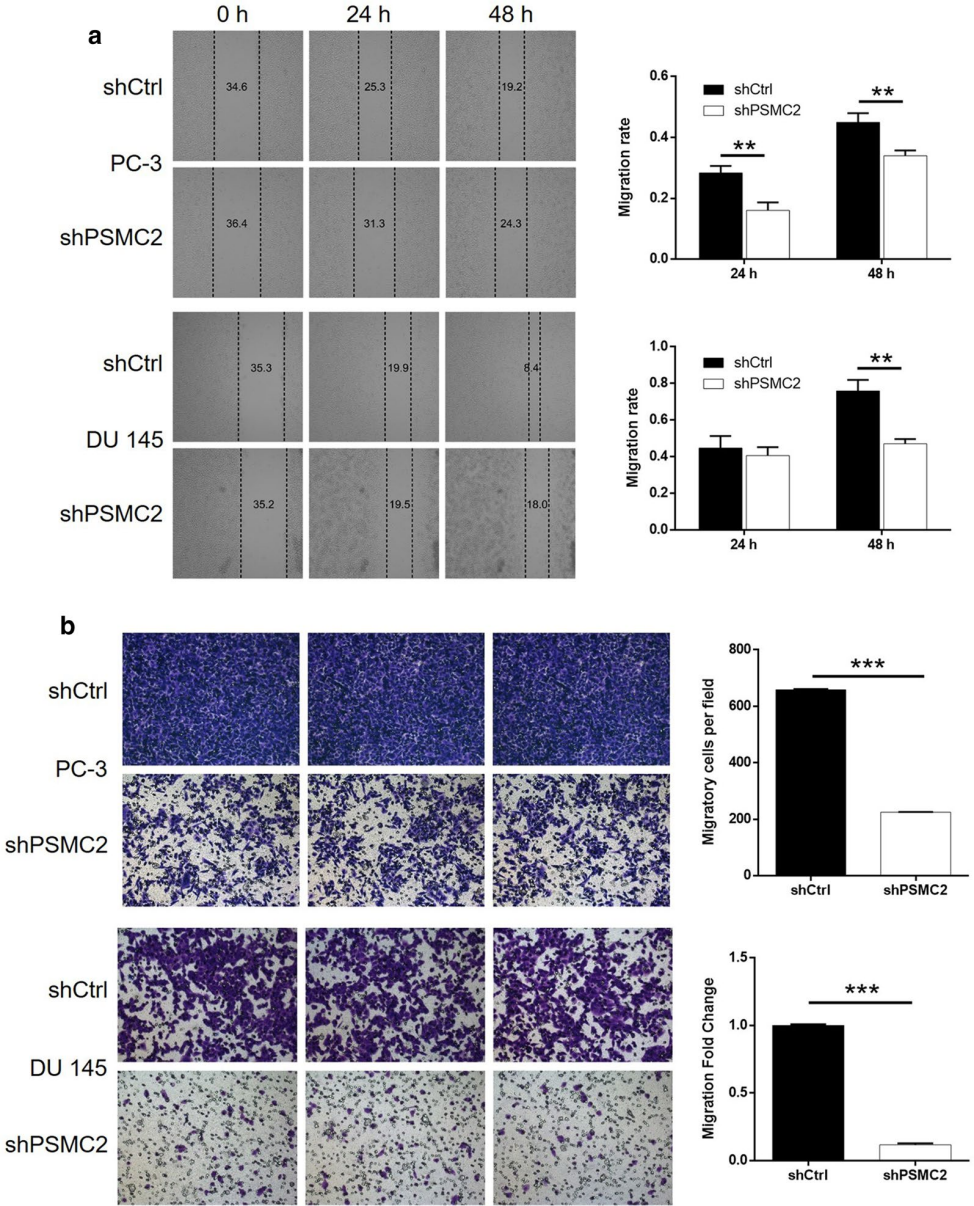
Knockdown of PSMC2 inhibited cell growth and cell migration. **a** Wound-healing and **b** Transwell assays were utilized to assess the effects of PSMC2 knockdown on cell migration of PC-3 and DU 145 cells. Results were presented as mean ± SD. *P < 0.05, **P < 0.01, ***P < 0.001

Next, we investigated the potential role of PSMC2 in the metastasis of prostate tumors and the migration of prostate cancer cells. Wound-healing assays showed that rate of migration for PC-3 and DU 145 cells when treated with shPSMC2 decreased significantly (*P* < 0.01, Fig. 4a), by 24% and 38%, respectively, after 24 h of culture. Transwell assays further showed that the knockdown of PSMC2 led to a significant inhibition of cell migration in both PC-3 and DU 145 cells (*P* < 0.01, Fig. 4b). These data demonstrated that PSMC2 depletion significantly inhibited the ability of prostate cancer cells to migrate.

### The knockdown of PSMC2 led to an inhibition of tumor growth in a mouse xenograft model

**Fig. 5:**
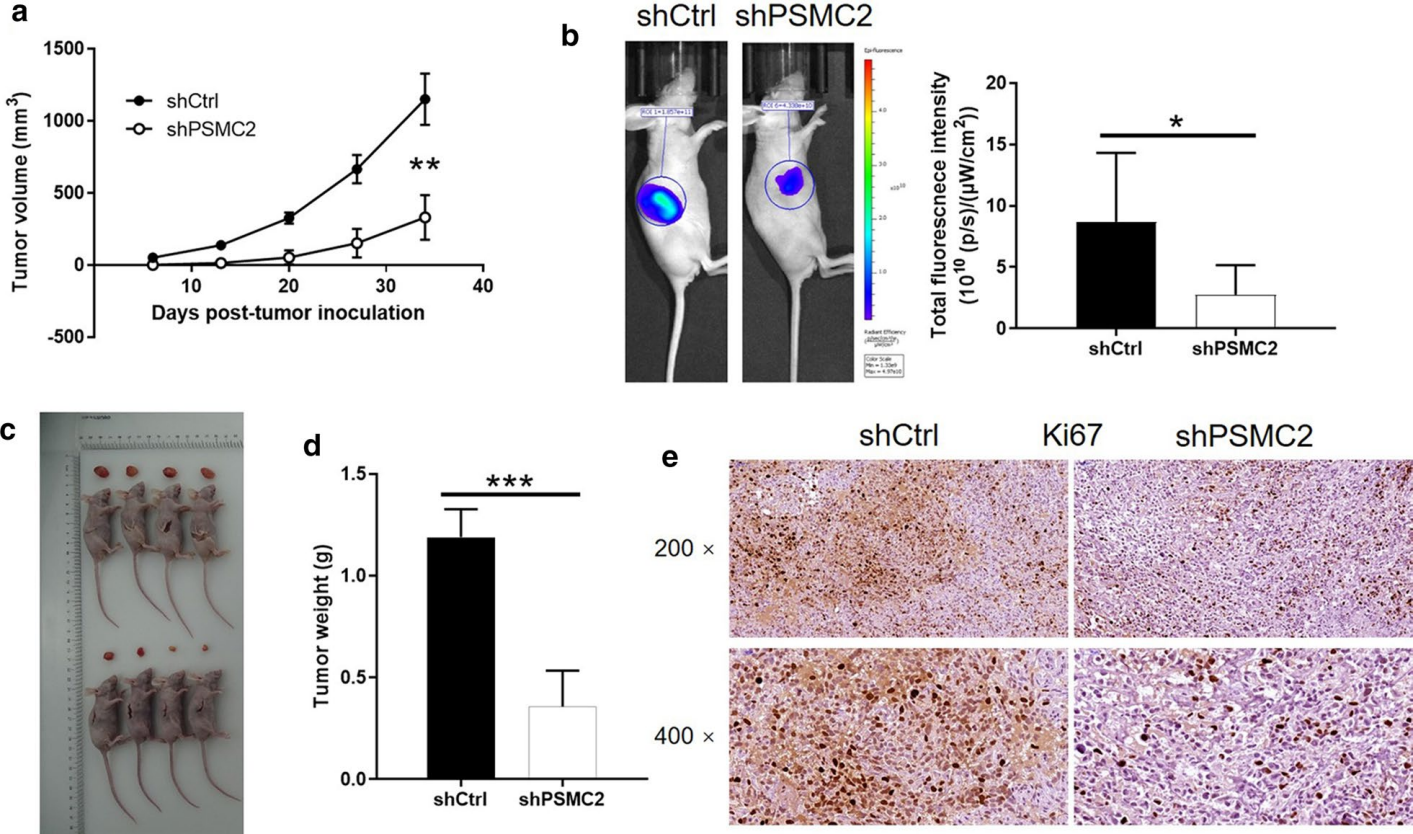
PSMC2 knockdown suppressed tumor growth in vivo. **a** The tumor volumes were measured throughout culture of animal models. **b** The bioluminescence intensity was obtained through injection of D-Luciferase before sacrificing the mice. **c** The photos of tumors were taken after the removal of tumors. **d** Tumor weights were measured after sacrificing the mice models. **e** Ki67 expression was detected by IHC in shCtrl and shPSMC2 xenografts. Results were presented as mean ± SD. *P < 0.05, **P < 0.01, ***P < 0.001

Next, we investigated the ability of PSMC2 knockdown to suppress the growth of tumors in vivo. PC-3 cells were injected subcutaneously with shCtrl and shPSMC2 and used to construct a mouse xenograft model. Animals treated with the shCtrl construct exhibited tumors that grew consistently in terms of volume. However, there were no discernable tumors in the mice that were injected with shPSMC2 (Fig. 5a). Bioluminescence imaging showed similar results following the injection of D-Luciferin (*P* < 0.001, Fig. 5b). 34 days after injection, the animals were sacrificed and the xenografts were removed and weighed; mice injected with shCtrl exhibited significantly larger tumors than that injected with shPSMC2 cells (*P* < 0.01, Fig. 5c and d). Moreover, it could also be observed that Ki67 expression, a representation of proliferative activity of tumors, was distinctly lower in shPSMC2 xenografts (Fig. 5e). Therefore, our in vivo studies demonstrated that the depletion of PSMC2 significantly inhibited the growth of prostate cancer cells.

### An analysis of the downstream mechanisms underlying the actions of PSMC2 on prostate cancer

**Fig. 6:**
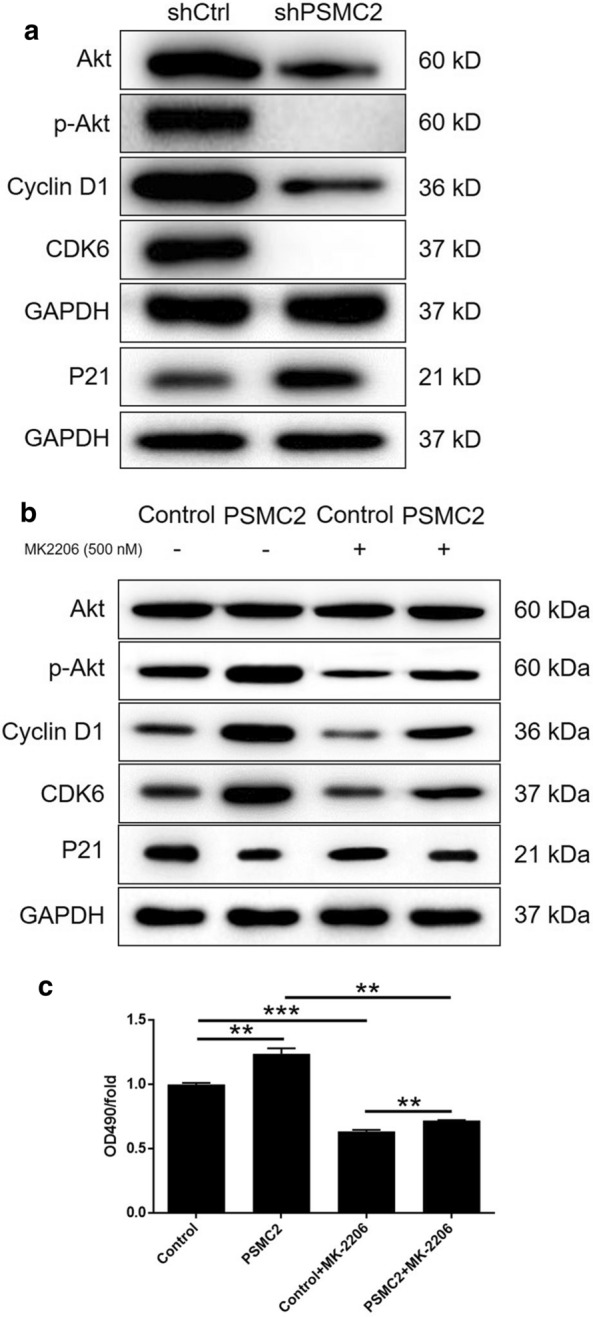
Exploration of mechanism underlying PSMC2-induced regulation of prostate cancer. **a** The expression levels of Akt, p-Akt, CDK6, Cyclin D1 and P21 detected by western blotting in PC-3 cells of shCtrl and shPSMC2 groups. **b** The expression levels of Akt, p-Akt, CDK6, Cyclin D1 and P21 detected by western blotting in PC-3 cells of Control and PSMC2 overexpression groups with or without treatment of MK-2206 (500 nM). **c** MTT assay was performed to assess the cell proliferation rate of PC-3 cells of Control and PSMC2 overexpression groups with or without treatment of MK-2206 (500 nM). Results were presented as mean ± SD. *P < 0.05, **P < 0.01, ***P < 0.001

In this part of the study, we investigated the mechanisms by which PSMC2 might regulate prostate cancer. To do this, we investigated the influence of PSMC2 depletion or the overexpression of several established cancer-related molecules in PC-3 cells by western blotting. As shown in Fig. 6a and b, the activity of the Akt pathway, along with the expression levels of Cyclin D1 and CDK6 were decreased in the shPSMC2 group, but were increased following the overexpression of PSMC2. Also, the upregulation of P21 in shPSMC2 cells was in consistent with previously mentioned results (Fig. 6a). More importantly, cells in which PSMC2 was, or was not, overexpressed by an Akt inhibitor (MK-2206) did not show suppression of the Akt pathway; instead, these cells showed downregulation in the levels of Cyclin D1 and CDK6. This indicated the potential involvement of the Akt/Cyclin D1/CDK6 pathway in the PSMC2-induced proliferation of prostate cancer (Fig. 6a and b). We also demonstrated that MK-2206 treatment significantly inhibited the proliferation of PC-3 cells, and partially reversed the effects of PSMC2 overexpression on the growth of PC-3 cells (Fig. 6c). Collectively, these results indicated that PSMC2 may promote the development of prostate cancer by regulating the Akt/Cyclin D1/CDK6 pathway.

## Discussion

The most common structural form of ubiquitin-mediated proteasomes in cells is the 26S proteasome, which contains a 20S core particle at the center and two 19S regulatory particles at either side [25, 26]. The 19S subunit of proteasome contains 19 protein components, each of which has its unique regulatory effect on the whole proteasome structure. PSMC2 is a necessary component of 19S subunit, which has the functions of ATP binding, nucleotide binding, nucleoside triphosphatase and hydrolase, and is mainly involved in regulating the selective degradation of intracellular proteins. In 2012, Nijhawan et al*.* identified 56 genes as CYCLOPS candidates during the study of cancer-specific vulnerabilities, which were enriched for spliceosome, proteasome and ribosome components, as well as essential genes in cell proliferation or survival. Among them, PSMC2 was reported to be the highest-ranked one with potential role in human cancers [20]. Song et al*.* investigated the role of PSMC2 in the development and metastasis of osteosarcoma, indicating the upregulation of PSMC2 in osteosarcoma tissues and elucidating the PSMC2 knockdown induced inhibition effects on cell proliferation, colony formation, cell motility and promotion of apoptosis as well as arrest of cell cycle in G2/M and/or S phase [23]. Moreover, Li et al*.* deepen the understanding of the role of PSMC2 in osteosarcoma through identifying it as the target of miR-630 in the promotion of cell proliferation, migration, invasion and correlation of poor prognosis [24]. Nevertheless, the relationship between PSMC2 and prostate cancer has not been reported and still remains unknown.

In this study, PSMC2 expression was enhanced in prostate cancer cell lines and tissues. PSMC2 overexpression was strongly associated with advanced Gleason grade and higher Gleason score. Beyond that, the in vitro experiments elucidated that the limited abilities of proliferation and colony formation of prostate cancer cells were particularly pronounced due to the inhibition of PSMC2. With respect to the results of wound-healing and transwell assays, the migration level of prostate cancer cells significantly decreased by PSMC2 knockdown. At the same time, our findings put forward the repressed cell apoptosis generated by PSMC2 knockdown as a result of the activation of apoptosis-related proteins including BIM, HSP27, IGF-II, p21, Survivin, sTNF-R1, sTNF-R2, TNF-β, TRAILR-4, XIAP. More importantly, it has come to light that PSMC2 knockdown attenuated tumor growth in vivo. The results of our study provided evidence for PSMC2 as a tumor promotor for prostate cancer.

Akt (also called as protein kinase B, PKB) is known for its great significance to regulate cell growth, proliferation, survival, as well as metabolism [27]. In agreement with previous reports indicating Akt as a target for the treatment of prostate cancer [28, 29], we also observed that knockdown of PSMC2 could decrease the activity of Akt pathway, especially the decline in its phosphorylation level. Conversely, PSMC2 overexpression observably stimulated the activation of Akt pathway, which could be restored to a normal level with the help of Akt inhibitor MK-2206. It was worth noting that cells with overexpressed PSMC2 showed the opposite phenotype, with the results of promoting cell growth, which was also found to be alleviated by the supplement of MK-2206. Based on our findings, we proposed that PSMC2 regulated prostate cancer cell partly by targeting Akt pathway. Cyclin D1 and CDK6, as the other two important regulators in cell cycle and cell proliferation [30–32], have been demonstrated to be highly expressed in prostate cancer and have enormous potential in wide various of cancer research and prognosis judgement [33–36]. Moreover, it has been reported before our study that both Cyclin D1 and CDK6 worked as downstream of Akt signaling pathway in the regulation of lung cancer and colorectal cancer [37, 38]. Corresponding to previous reports, our results have pointed to an obvious downregulation of both Cyclin D1 and CDK6 upon knockdown of PSMC2. Different from knockdown of PSMC2, we obtained the conversed results when PSMC2 overexpression. Subsequently, upregulation of Cyclin D1 and CDK6 was also partially reversed by MK-2206, which served as an evidence for the involvement of Akt/Cyclin D1/CDK6 pathway in prostate cancer. Albeit these results, the underlying mechanism of the regulatory effects of PSMC2 on the development and metastasis of prostate cancer is still poorly understood and would be the focus of next research.

## Conclusion

In conclusion, this study was the first to verify the promotor role played by PSMC2 in development and metastasis of prostate cancer. It was also the first study to demonstrate that high expression of PSMC2 was correlated with more advanced Gleason grade and higher Gleason score. Knockdown of PSMC2 gave rise to the blocked development and metastasis of prostate cancer, which was probably resulted from regulating Akt/Cyclin D1/CDK6 signalling pathway. Our experimental data might provide a strategy for targeting with the PSMC2/Akt/Cyclin D1/CDK6 signalling pathway interaction as a novel therapeutic application to treat prostate cancer patients.

## Data Availability

The datasets during and/or analysed during the current study available from the corresponding author on reasonable request.
